# Developing a resiliency model for survival without major morbidity in preterm infants

**DOI:** 10.1038/s41372-022-01521-3

**Published:** 2022-10-11

**Authors:** Martina A. Steurer, Kelli K. Ryckman, Rebecca J. Baer, Jean Costello, Scott P. Oltman, Charles E. McCulloch, Laura L. Jelliffe-Pawlowski, Elizabeth E. Rogers

**Affiliations:** 1 Department of Pediatrics, University of California San Francisco, San Francisco, CA USA; 2 Department of Epidemiology and Biostatistics, University of California San Francisco, San Francisco, CA USA; 3 Department of Epidemiology, College of Public Health, University of Iowa, Iowa City, IA USA; 4 California Preterm Birth Initiative, University of California San Francisco, San Francisco, CA USA

**Keywords:** Risk factors, Outcomes research

## Abstract

**Objective:**

Develop and validate a resiliency score to predict survival and survival without neonatal morbidity in preterm neonates <32 weeks of gestation using machine learning.

**Study design:**

Models using maternal, perinatal, and neonatal variables were developed using LASSO method in a population based Californian administrative dataset. Outcomes were survival and survival without severe neonatal morbidity. Discrimination was assessed in the derivation and an external dataset from a tertiary care center.

**Results:**

Discrimination in the internal validation dataset was excellent with a c-statistic of 0.895 (95% CI 0.882–0.908) for survival and 0.867 (95% CI 0.857–0.877) for survival without severe neonatal morbidity, respectively. Discrimination remained high in the external validation dataset (c-statistic 0.817, CI 0.741–0.893 and 0.804, CI 0.770–0.837, respectively).

**Conclusion:**

Our successfully predicts survival and survival without major morbidity in preterm babies born at <32 weeks. This score can be used to adjust for multiple variables across administrative datasets.

## Introduction

In infants born preterm, gestational age (GA) is the best indicator of maturity and is a strong driver of survival and other health and neurodevelopmental outcomes [1]. Several other predictors influence outcomes in this vulnerable patient population. In their landmark paper, Tyson and colleagues showed that consideration of birth weight, sex, antenatal steroid administration, and multiple gestation improved prediction of survival of extremely low gestational age infants [2]. Several prediction models have subsequently been developed to predict mortality and morbidity in very preterm neonates including a variety of maternal, antenatal, and postnatal candidate predictors with variable predictive performance [3, 4].

Risk assessment and outcome prediction in extremely preterm neonates is important for multiple reasons. Understanding an infant’s range of potential outcomes can inform counseling and medical decision making, both antenatally when making shared decisions around the provision of intensive care at delivery as well as postnatally if complications develop [5]. Another important goal of prediction models includes their use in benchmarking and comparing performance across centers to guide continuous quality improvement in care practices [6]. Finally, prediction models can help ensure that both care practices and outcomes are observed to be equitable in order to reduce the impact of discrimination and structural racism [7].

It has been shown that race/ethnicity as well as certain sociodemographic factors are associated with a variety of long-term outcomes in very preterm neonates [8, 9]. Some of the previously described prediction models include race/ethnicity and/or sociodemographic factors [4]. This approach, however, assumes that the models are performing equally across all racial, ethnic, and sociodemographic groups, which should not be assumed. It may also limit our ability to adequately address risk and resiliency specifically in communities most in need.

Interestingly, all currently existing survival and major morbidity prediction models for infants born preterm have defined their prediction around “risk factors.” Such an approach may encourage a focus by care providers on deficits rather than on strengths that may foster better outcomes. Resilience can be conceptualized as a positive adaptation to an experienced adversity. This concept may deepen our understanding of the variation in outcomes after the adverse experience of preterm birth [10–12]. Focusing on resilience rather than risk in predictive modeling may shift our framework from identifying deficits to promotive factors [10]. This frameshift has also been prioritized by families experiencing preterm birth.

The goal of this study was to develop a prediction model for survival without major morbidity in preterm infants born at <32 weeks using a resiliency frame. Machine learning was used to build a resiliency score with a focus on maternal, perinatal, and neonatal factors known at the time of birth. The model was externally validated. Performance was assessed across race/ethnic and sociodemographic subgroups to ensure validity across diverse populations.

## Methods

Our primary dataset was drawn from all California live born infants between 2011 and 2017. Birth and death certificates (up to 12 months of age) from California Vital Statistics were linked to the database maintained by the California Office of Statewide Health Planning and Development (OSHPD). This dataset was randomly divided into a training dataset (2/3 of the data) and an internal validation dataset (1/3 of the data). We also externally validated the model using a retrospective cohort derived from the Iowa Perinatal Health Research Collaborative (IPHRC) database.

The primary OSHPD dataset included detailed information on maternal and infant characteristics from hospital discharge records from one year prior to birth to one year of age. The data included diagnosis and procedure codes based on the *International Classification of Diseases, 9*th *and 10*th *Revision, clinical modification* (ICD-CM). GA was determined by best obstetric estimate from ultrasound and/or last menstrual period. This dataset has been used in multiple studies examining birth and neonatal outcomes [13–17]. All linked singleton liveborn infants with GA < 32 weeks without major congenital or chromosomal anomalies were included in this study (ICD-9 and 10 codes used to define major congenital or chromosomal anomalies are listed in Supplemental Table 1).

The primary outcome was survival to 1 year of age (determined by death certificate or death as the hospital discharge status). The secondary outcome was a composite of survival without any major neonatal morbidity. Major neonatal morbidity was determined from ICD-9 and ICD-10 CM codes. These included bronchopulmonary dysplasia (BPD, ICD-9 770.7, ICD-10 P27.1), necrotizing enterocolitis (NEC, ICD-9 777.5, ICD-10 P77), intraventricular hemorrhage > grade II (IVH, ICD-9 772.13 and 772.14, ICD-10 P52.2), periventricular leukomalacia (PVL, ICD-9 779.7, ICD-10 P91.2), and retinopathy of prematurity (ROP) > stage 2. To define ROP > stage 2 we used diagnostic or procedure codes (ICD-9 362.25-7 or 14.2, 14.5, 14.7 14.9, ICD-10 H35.14-6 or any procedure code for surgery on retina or choroid plexus).

The data was randomly divided into a training and validation dataset as described above. The model was developed using the training sample only. Maternal, perinatal, and neonatal covariates that have been significantly associated with neonatal outcomes in prior studies were considered for inclusion in the model (Supplemental Table 2).

We fit a logistic regression model to the outcomes. Due to the large number of potential covariates, the model was built using the least absolute shrinkage and selection operator (LASSO). The LASSO is a logistic regression method that is able to accommodate large numbers of predictors in a statistically principled way to reduce model complexity and avoid over-fitting the prediction model. It involves penalizing the absolute size of the regression coefficients with the consequence that regression coefficients are reduced in absolute size and variables that do not strongly predict the outcome have the coefficients set to zero, eliminating them from the model. Cross-validation (within the training data) was used to select the optimal weighting parameter (lambda) and thus the final model. The resiliency score for a specific observation consists of the sum of all the beta coefficients based on the state of the observed covariates. In order to convert the resiliency score to a survival probability, the constant of the model is added to the score and the following formula can be used: survival probability = e^(constant + resiliency score)^/(1 + e^(constant + resiliency score)^).

Discrimination was assessed in the internal and external validation samples by calculating c-statistics and receiver operator characteristic curves (ROCs) for the resiliency score and both of our outcomes. Calibration was assessed in the primary dataset by dividing the validation sample into deciles based on the resiliency score values and comparing predicted and observed survival and survival without major morbidity rates for each decile of the validation sample.

For each subject we calculated the predicted outcome probabilities based on the model. We then stratified the sample by gestational age and found the smallest and the largest observed predicted outcome probability in this group. We then stratified the sample by race/ethnicity, insurance status, and maternal education to assess the resiliency score performance in different race/ethnic and sociodemographic groups. For that purpose, we calculated the observed outcome probability and the predicted outcome probability for each subgroup. The predicted outcome probability was calculated by finding the mean value of all predicted outcome probabilities in the respective subgroup.

We also performed external validation in a retrospective cohort derived from the Iowa Perinatal Health Research Collaborative (IPHRC) database, a resource composed of linked maternal and newborn electronic health record (EHR) data for deliveries occurring at a single tertiary care unit. EHR data within this database were provided by the Institute for Clinical and Translational Science (ICTS) Bioinformatics Core at the University of Iowa. The validation dataset included 1347 deliveries at <32 weeks gestation born between January 1, 2012 and December 31, 2019. We excluded 261 deliveries that were missing either the maternal or delivery medical record, 4 newborns transferred out of the facility during the first day of life, 13 fetal demises, 294 twin or higher order multiples, 13 infants born to mothers already included in the dataset with a previous pregnancy, and 86 newborns with a major congenital anomaly as defined in Supplemental Table 3.

The primary and secondary outcomes were defined in the same manner as in the derivation dataset. Maternal, perinatal, and neonatal characteristics were defined by ICD9 and ICD10 codes as outlined above (Supplemental Table 2) examining infant codes up to 365 days after birth and maternal codes between 280 days before delivery through 42 days after delivery. Maternal body mass index (BMI) was derived either directly from a reported BMI in the EMR or by calculating BMI using weight and height, using the earliest recorded BMI or height and weight during the antenatal period. Mode of delivery was recorded directly from the medical record. Resiliency scores for survival and survival without major morbidity were calculated for the Iowa population using the coefficients from the model developed in the California dataset and AUCs were calculated to assess discrimination in the validation dataset.

All analyses were performed by using STATA version 16.1 (Stata Statistical Software: Release 16. College Station, TX: StataCorp LP). The use of the OSHPD database was approved by the Committee for the Protection of Human Subjects within the California Health and Human Services Agency, the use of the Iowa dataset was approved for a waiver of consent by the University of Iowa Institutional Review Board (IRB number: 202007280).

We utilized California’s Office of Statewide Health Planning and Development database (OSHPD). The data use agreement with the OSHPD prohibits distribution of any patient-level data; thus, the data used for this study are not made publicly available. Data can be requested from OSHPD (https://www.oshpd.ca.gov/HID/HIRC/index.html) by qualified researchers for a fee. Similarly, the data use agreement of the Iowa clinical database does not allow to share patient level data. All other analytic methods and study materials are available upon reasonable request from the corresponding author.

## Results

We identified 21,483 preterm infants born alive <32 weeks of gestation in California from 2011–2017. Mortality in the entire dataset was 13.5% (2896/21483), 18.1% survived with neonatal morbidity (3884/21483) and 68.4% survived without morbidity (14,703/21,483). The training dataset consists of 14,322 infants and the validation dataset of 7161 infants, respectively. Maternal, perinatal, and neonatal characteristics for the training dataset are shown in Table 1.

**Table 1:** Characteristics of the derivation dataset (*n* = 14,322).

	Survived (*n* = 12398)	Died (*n* = 1924)
*Maternal covariates*		
Maternal age, median (IQR)	30 (25–35)	29 (24–34)
Preeclampsia, *n* (%)	3064(24.7%)	201(10.5%)
Hypertension, *n* (%)	720 (5.8%)	111 (5.8%)
Diabetes, *n* (%)	2266 (18.3%)	224 (11.6%)
BMI, median (IQR)	26.2 (22.4–31.2)	26.6 (22.6–31.9)
Smoking, *n* (%)	739 (6.0%)	135 (7.0%)
Drug abuse, *n* (%)	780(6.3%)	129 (6.7%)
Infection during pregnancy, *n* (%)	2886 (23.3%)	389 (20.2%)
*Perinatal covariates*		
Placental abruption, *n* (%)	1975(15.9%)	350 (18.2%)
Uterine rupture, *n* (%)	37 (0.3%)	3 (0.2%)
PROM, *n* (%)	3784 (30.5%)	670 (34.8%)
Preterm labor, *n* (%)	6268 (50.6%)	994 (51.7%)
C-section, *n* (%)	8077(65.2%)	879 (45.7%)
Oligohydramnios, *n* (%)	671(5.4%)	110 (5.7%)
Polyhydramnios, *n* (%)	120 (1.0%)	50 (2.6%)
*Neonatal factors*		
Female sex, *n* (%)	5760 (46.5%)	835 (43.4%)
GA, median (IQR)	29 (27–31)	24 (22–25)
Z-score for BW, median (IQR)	0.17 (−0.42 to 0.73)	0 (−0.62 to 0.56)

*BMI* body mass index, *PROM* premature rupture of membranes, *GA* gestational age, *BW* birth weight, *IQR* interquartile range.

After applying the LASSO method for survival, the cross validated lambda was 0.00075. Supplemental Fig. 1a shows the cross-validation plot. Out of the 30 covariates, 29 were retained in the model: …maternal age, maternal BMI, sex, gestational age and z-score for BW, vaginal delivery, absence of preeclampsia, hypertension, diabetes, smoking, drug abuse, infection, abruption, uterine rupture or PPROM, oligohydramnios, or polyhydramnios. The only factor not included in the model is “no preterm labor.” Similarly, after applying the LASSO method for survival without severe morbidity, the cross validated lambda was 0.00032. Supplemental Fig. 1b shows the cross-validation plot. All 30 covariates were retained in the final model. Table 2 shows the beta coefficient for each covariate or covariate level from the LASSO model for survival and survival without severe morbidity. Covariate categories with a listed value of 0 are the reference category. Covariates or covariate categories with a negative beta coefficient reduce the resiliency score and are negatively associated with the specified outcomes, while covariates or covariate categories with a positive beta coefficient are positively associated with survival or survival without severe morbidity.

**Table 2:** Prediction model for survival neonates < 32 weeks gestational age in training dataset (*n* = 14,322).

	Survival	Survival without significant morbidity
	Beta coefficient	Beta coefficient
*Constant*	2.801	1.106
*Maternal covariates*		
Maternal age, years		
≤18	−0.067	−0.020
>18 to <35	0	0.027
≥35	0.001	0
No preeclampsia	−0.188	−0.118
No hypertension	−0.0507	0.005
No diabetes	−0.062	0.053
BMI		
Underweight (<18.5)	−0.040	−0.030
Normal weight (18.5–24.9)	0	−0.029
Overweight (25–30)	0.027	0.011
Obese (>30)	−0.005	0
No smoking	0.005	0.041
No drug abuse	0.044	0.030
No infection	−0.021	−0.027
*Perinatal covariates*		
No abruption	0.061	0.012
No uterine rupture	0.011	−0.005
No PROM	−0.035	−0.069
No preterm labor	NA	−0.046
Vaginal delivery	−0.036	0.039
No Oligohydramnios	0.029	0.021
No Polyhydramnios	0.248	0.165
*Neonatal factors*		
Male sex	−0.130	−0.097
GA		
22	−0.968	−0.773
23	−0.704	−0.608
24	−0.530	−0.452
25	−0.328	−0.29
26	−0.218	−0.179
27	0	0
28	0.10	0.199
29	0.220	0.416
30	0.411	0.691
31	0.608	1.0
Z-score for BW		
<−1	−0.419	−0.355
−1 to < 0	−0.187	−0.156
0 to 1	0.0	0.0
>1	0.069	1.166

*BMI* body mass index, *PROM* premature rupture of membranes, *GA* gestational age, *BW* birth weight.

Calculation of resiliency score: add all beta coefficient based on the specific covariates for the baby (for example for 30weeks GA add 0.411, for no PROM subtract −0.035).

Calculation of predicted survival: use the following formula to convert the resiliency score to predicted survival e^(constant + resiliency score)^/(1 + e^(constant + resiliency score)^). The constant is displayed at the top of the table. For example, if the resiliency score is −0.5, then the predicted survival = e^(2.801-0.5)^/(1 + e^(2.801-0.5)^)=90.9%.

Discrimination of the model in the internal validation dataset was excellent with a c-statistic of 0.895 (95% CI 0.882–0.908) for survival and 0.867 (95% CI 0.857–0.877) for survival without severe neonatal morbidity, respectively (Supplemental Fig. 2). Calibration of both models was also excellent with very similar observed and predicted rates for both outcomes (Supplemental Table 4).

**Fig. 1:**
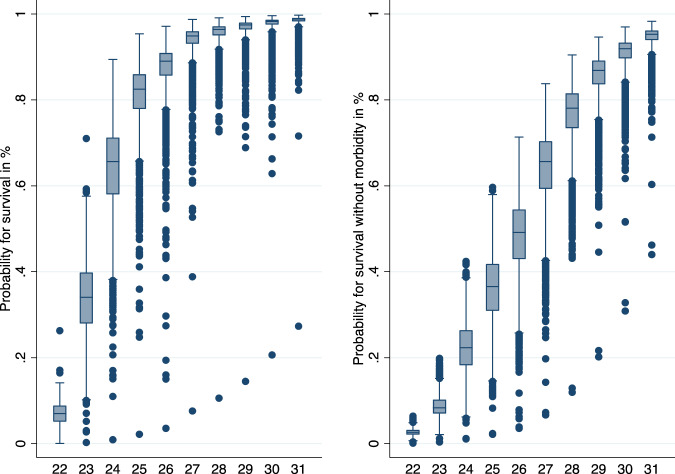
The boxplots for observed predicted probability for survival and survival without morbidity by gestational age.

The strong predictive value of GA can be seen in the wide range of values for the beta coefficients for GA categories: for survival, the beta coefficient is −0.968 for GA of 22 weeks and +0.608 for GA of 31 weeks, and for survival without major neonatal morbidity, the beta coefficient for GA of 22 weeks is −0.773 while it is +1.0 for GA of 31 weeks. However, the other covariates also contribute significantly to the model. Supplemental Table 5 and Fig. 1 show the relative importance of all the other covariates by presenting the smallest and largest outcome range based on the model stratified by each week of GA.

Supplemental Table 6 shows the observed and expected outcome probabilities for racial and ethnic groups as well as for groups defined by different sociodemographic factors. The observed and expected outcome probabilities closely approximate each other for each of these subgroups.

With regards to external validation, the baseline characteristics of the Iowa dataset are presented in Table 3. Mortality in this dataset was 5.6% (38/676); 46.9% (317/676) survived with neonatal morbidity and 47.5% (321/676) survived without morbidity. However, the proportion of survived with neonatal morbidity dropped to 4.3% (29/676) when BPD was excluded from the neonatal morbidity definition. Discrimination of the model in the external validation dataset remained excellent with a c-statistic of 0.817 (95% CI 0.741-0.893) for survival and 0.804 (95% CI 0.770-0.837) for survival without severe neonatal morbidity, respectively. Calibration in the external validation dataset is shown in Table 4 for the morbidity definition including and excluding BPD.

**Table 3:** Characteristics of the external validation dataset (*n* = 676).

	Survived (*n* = 638)	Died (*n* = 38)
*Maternal covariates*		
Maternal age, median (IQR)	28.9 (24.7 to 33.1)	30.3 (25.8 to 34.3)
Preeclampsia, *n* (%)	183 (28.7)	<5
Hypertension, *n* (%)	143 (22.4)	6 (15.8)
Diabetes, *n* (%)	86 (13.5)	6 (15.8)
BMI, median (IQR)^a^	29.2 (25.2 to 34.8)	32.0 (25.7 to 37.1)
Smoking, *n* (%)	104 (16.3)	5 (13.2)
Drug abuse, *n* (%)	45 (7.1)	<5
Infection during pregnancy, *n* (%)	120 (18.8)	<5
*Perinatal covariates*		
Placental abruption, *n* (%)	107 (16.8)	6 (15.8)
Uterine rupture, *n* (%)	<5	<5
PPROM, *n* (%)	240 (37.6)	20 (52.6)
Preterm labor, *n* (%)	399 (62.5)	27 (71.1)
C-section, *n* (%)	401 (62.9)	16 (42.1)
Oligohydramnios, *n* (%)	52 (8.2)	<5
Polyhydramnios, *n* (%)	9 (1.4)	<5
*Neonatal factors*		
Female sex, *n* (%)	295 (46.2)	17 (44.7)
GA, median (IQR)	28 (26 to 30)	24 (22 to 26)
Z-score for BW, median (IQR)	0.13 (−0.49 to 0.64)	0.14 (−0.49 to 0.59)

^a^missing BMI for 44 mothers.

*BMI* body mass index, *PROM* premature rupture of membranes, *GA* gestational age, *BW* birth weight.

**Table 4:** Calibration in the external validation sample for survival and survival without major morbidity including and excluding BPD (in deciles).

	Survival		Survival without major morbidity (including BPD)		Survival without major morbidity (excluding BPD)	
*n*	Observed (%)	Predicted (%)	Observed (%)	Predicted (%)	Observed (%)	Predicted (%)
68	72.1	86.6	5.9	61.5	69.1	61.5
69	91.3	90.4	5.9	68.6	91.2	68.6
66	93.9	92.1	17.9	72.7	76.1	72.7
68	92.7	93.2	30.9	76.9	94.1	76.9
67	98.5	94.2	58.0	80.3	95.7	80.3
68	100.0	94.8	62.1	83.2	93.9	83.2
68	100.0	95.3	73.5	85.9	92.7	85.9
67	98.5	95.9	73.1	88.1	95.5	88.1
69	98.6	96.4	76.8	89.7	94.2	89.7
66	98.5	97.0	71.2	93.5	98.5	93.5

*BPD* bronchopulmonary dysplasia.

## Discussion

Using maternal, perinatal, and neonatal predictors readily available in most administrative databases at the time of birth, we built a model based on resiliency score with excellent discrimination and calibration to predict survival and survival without neonatal morbidity in neonates <32 weeks of gestational age. This prediction model performed well in an external validation cohort. While gestational age had the strongest influence on the resiliency score, the other included predictors increased the predictive ability of the model. The resiliency score performed well in all race/ethnic and sociodemographic subgroups.

A wide variety of predictive models for survival and survival without severe morbidity after preterm birth have been developed [2–4, 18]. Some of these models are parsimonious and others incorporate a wide variety of predictors. Like previous models, the main driver for successful prediction in our model is GA at birth, as can be clearly seen by the variability of the beta coefficients. Importantly, despite GA being the strongest predictor of survival and survival without severe morbidity, other factors contribute and should not be ignored. In our prediction model, we show how outcome predictions vary widely for the same GA based on other predictors, validating the importance of these factors. For example, a neonate born at 23 weeks with the highest resiliency score, or most favorable covariates, had a predicted survival chance of 66.7%, while the neonate born at the same GA with the lowest resiliency score, or least favorable covariates, had a predicted chance of survival of 0.5%.

Our model adds to the existing literature on neonatal outcome prediction in a few novel ways. First, we used machine learning, more specifically the LASSO method, to develop our model. The main benefit of the LASSO method compared to traditional logistic regression is the ability of the LASSO method to select relevant variables. It is superior to forward or backward selection often used in logistic regression and avoids overfitting by shrinking the coefficients [19]. Second, the main advantage of this score is that it incorporates many variables that are available in the different administrative datasets available. This score can be used to adjust for several important maternal and neonatal factors and may make comparison between databases more streamlined.

The focus of our model on resilience or promotive factors, rather than risks or maladaptive factors, is a frameshift from previous algorithms focused on risk. Preterm birth has increasingly been understood to be an adverse event biologically for the infant, after which both adaptive and maladaptive processes can occur to render an infant resilient or at risk [10, 12] There have been previous studies focused specifically on resiliency of preterm infants, but these have focused on longer term outcomes at transition to home or in early school age, rather than in the immediate neonatal hospitalization [11, 20]. Additionally, caregivers of preterm infants have increasingly called for the use of strength-based frameworks, such as the use of hope and resilience, both for antenatal counseling and in understanding long term prognosis of their infants [21]. For our model to fit the needs of caregivers and bedside clinicians as critical stakeholders in the outcomes, we chose this specific frameshift from risk to resilience. We would like to acknowledge though that resiliency is just the opposite of risk, and survival is just the opposite of mortality. If we were to develop a traditional risk score, the results with regards to discrimination and calibration would have been the same.

We intentionally did not include race/ethnicity or socioeconomic risk factors in our model. There is increasing recognition that inclusion of race and ethnicity into prediction algorithms might lead to bias and can exacerbate health care disparities [22]. For example, the calculator for successful vaginal birth after cesarean section (VBAC) included race/ethnicity. A Hispanic or Black woman with the same medical characteristics as a white woman had a much lower predicted success rate for VBAC [23]. In this example, if providers are influenced by concerns over perceived risk, they may be less likely to offer a trial of labor to women with low VBAC scores, and the race-based correction in the VBAC calculator may exacerbate racial disparities [23]. It is important to recognize that race and ethnicity reflect social constructs rather than a biological truth wherein biological definitions of race have been challenged by findings of greater genetic variation within rather than between groups based on skin color [24]. Several recent studies highlight the importance of minimizing racial bias in prediction models [25, 26]. Similarly, socioeconomic factors have been shown to be associated with poor clinical outcomes [27]. However, this association is often mediated through medical risk factors and predictors as well as structural discrimination; as such, we elected to not include those into the model. We present model performance in different race/ethnic and socioeconomic groups and found that it performed well across groups. This is an important finding and will allow this scoring to be used across subpopulations thereby minimizing biased approaches to care in different race/ethnic and socioeconomic groups.

A significant strength of this study is the use of an external validation dataset. While our internal validation avoided overfitting of the model, the external validation shows that this model can discriminate well in other populations. The calibration in the Iowa dataset identified interesting issues. BPD has historically been a complicated morbidity to capture accurately in administrative datasets based on differing definitions. The BPD ICD9 and 10 codes are used significantly more often in the Iowa dataset compared to California due to local definitions of BPD, leading to different rates of neonatal morbidity in the two cohorts. This led to overestimation of survival without neonatal morbidity in the calibration table. After excluding BPD from the morbidity definition, the neonatal morbidity is extremely low in the Iowa cohort at just 4.3%, and therefore, the model underestimates the survival without major morbidity. The truth is somewhere in between as some of the infants in this cohort will have long-term pulmonary morbidity. This example shows the importance of understanding local context and the potential need to recalibrate the model if it is used across different populations or with variable definitions of important outcomes.

As the above paragraph illustrates, one of the main weaknesses of this study is that it relies on ICD9 and ICD10 codes, which can lead to misclassification. BPD has historically been a complicated morbidity to capture accurately in administrative datasets. Coders with limited medical knowledge often determine that diagnosis leading to an overestimation in certain datasets. For example, BPD is often used as a diagnosis for any infant with respiratory distress and prolonged respiratory support. Additionally, the score can only be used in datasets with the available variables which might reduce its potential for broad application.

Our model using resiliency score is shown here to successfully predict survival and survival without major morbidity in preterm babies born at <32 weeks. In addition, it can be used across different epidemiological settings and race/ethnic and sociodemographic subpopulations. A resiliency score can be used and be helpful in clinical settings and in antenatal and postnatal counseling with a focus on protection rather than risk.

## Supplementary information

supplemental Table 1

supplemental Table 2

supplemental Table 3

supplemental Table 4

supplemental Table 5

supplemental Table 6

supplemental Figure 1

supplemental Figure 2

## Data Availability

The data use agreement with the OSHPD prohibits distribution of any patient-level data; thus, the data used for this study are not made publicly available. Data can be requested from OSHPD (https://www.oshpd.ca.gov/HID/HIRC/index.html) by qualified researchers for a fee. Similarly, the data use agreement of the Iowa clinical database does not allow to share patient level data. All other analytic methods and study materials are available upon reasonable request from the corresponding author.
