# Supplementary material for: Developing a resiliency model for survival without major morbidity in preterm infants
Source: J Perinatol. 2022 Oct 11;43(4):452–7. doi: 10.1038/s41372-022-01521-3 (PMC10079534; doi:10.1038/s41372-022-01521-3)
Supplement: Supplementary file 1 — supplemental Table 1 [file 41372_2022_1521_MOESM1_ESM.docx]

Supplemental Table 1: Definition of Major Birth Defects and chromosomal anomalies used in California.

| **Birth Defect** | **ICD-9-CM Codes** | **ICD-10-CM Codes** |
| --- | --- | --- |
| **CNS** |  |  |
| **Anencephalus** | 740.0x — 740.1x | Q00.x |
| **Spina bifida without**  **anencephalus** | 741.0x, 741.9x | Q05.x |
| **Encephalocele** | 742.0x | Q01.x |
| **Holoprosencephaly** | 742.2x | Q04.0.x, Q04.1x, Q04.2x, Q04.3x |
| **Congenital hydrocephalus** | 742.3x | Q03.x |
| **Eye** |  |  |
| **Anophthalmia/microphthalmia** | 743.0x, 743.1x | Q11.x |
| **Congenital cataract** | 743.3x | Q12.0x |
| **Coloboma** | 743.4x | Q13.x |
| **Ear** |  |  |
| **Anotia/microtia** | 744.01x, 744.23x | Q16.0x, Q17.2x |
| **Cardiovascular** |  |  |
| **Aortic valve stenosis** | 746.3x | Q23.0x |
| **Endocardial cushion defects** | 745.6x | Q21.2x |
| **Coarctation of the aorta** | 747.10x | Q25.1x |
| **Common truncus** | 745.0x | Q20.0x |
| **Transposition of great vessels** | 745.1x | Q20.3x |
| **Ebstein anomaly** | 746.2x | Q22.5x |
| **Hypoplastic left heart syndrome** | 746.7x | Q23.4x |
| **Coarctation of aorta** | 747.1x | Q25.2x, Q25.4x |
| **Pulmonary valve atresia and stenosis** | 746.0x | Q22.0x, Q22.2x |
| **Single Ventricle** | 745.3x | Q20.4x |
| **Tetralogy of Fallot (TOF)** | 745.2x | Q21.3x |
| **Anomalies of great veins** | 747.4x | Q26.2x |
| **Tricuspid value atresia and stenosis** | 746.1x | Q22.4x |
| **Pulmonary** |  |  |
| **Choanal atresia** | 748.0x | Q30.0x |
| **Congenital cystic lung** | 748.2x | Q33.0x |
| **Agenesis, hypoplasia, and dysplasia of the lung** | 748.5 | Q33.3 |
| **Orofacial** |  |  |
| **Cleft lip with cleft palate** | 749.2x | Q37.x |
| **Cleft lip alone (without cleft palate)** | 749.1x | Q36. x |
| **Cleft palate alone (without cleft lip)** | 749.0x | Q35.x |
| **Gastrointestinal** |  |  |
| **Anomalies of gallbladder bile ducts and liver (including biliary atresia)** | 751.6x | Q44.x |
| **Esophageal atresia/tracheoesophageal fistula** | 750.3x | Q39.0x — Q39.4x |
| **Congenital hypertrophic pyloric stenosis** | 750.5x | Q40.0x |
| **Rectal and large intestinal atresia/stenosis** | 751.2x | Q42.x |
| **Small intestinal atresia/stenosis** | 751.1x | Q41.x |
| **Hirschsprung disease and other congenital functional disorders of colon** | 751.3x | Q43.1x |
| **Genitourinary** |  |  |
| **Bladder exstrophy** | 753.5x | Q64.1x |
| **Congenital Posterior Urethral Valves** | 753.6x | Q64.2x |
| **Hypospadias** | 752.6x | Q54.x |
| **Indeterminate sex and pseudohermaphroditism** | 752.7x | Q56.x |
| **Renal agenesis/hypoplasia** | 753.0x | Q60.x |
| **Obstructive genitourinary defects** | 753.2x, 753.6x | Q62.x, Q64.3x |
| **Musculoskeletal** |  |  |
| **Anomalies of skull and face bones (including craniosynostosis)** | 756.0x | Q75.x |
| **Anomalies of diaphragm (including diaphragmatic hernia)** | 756.6x | Q79.0x, Q79.1x |
| **Anomalies of abdominal wall (including gastroschisis and omphalocele)** | 756.7x | Q79.2x, Q79.3x, Q79.4x, Q79.5x |
| **Limb deficiencies (reduction defects)** | 755.2x, 755.3x, 755.4x | Q71.x, Q72.x |
| **Chondrodystrophy/ Osteodystrophies** | 756.4x, 756.5x | Q78.x |
| **Chromosomal** | 758.x | Q9x |

NOTE: x means included all decimal places after this (e.g. for Encephalocele: code is 742.0x, so individuals with 742.0, 742.01, 742.02, 742.03, 742.04, etc. would be counted as having this major congenital anomaly)
