# Supplementary material for: Developing a resiliency model for survival without major morbidity in preterm infants
Source: J Perinatol. 2022 Oct 11;43(4):452–7. doi: 10.1038/s41372-022-01521-3 (PMC10079534; doi:10.1038/s41372-022-01521-3)
Supplement: Supplementary file 2 — supplemental Table 2 [file 41372_2022_1521_MOESM2_ESM.docx]

Supplemental Table 2: ICD 9 and 10 codes used to define maternal, perinatal and neonatal variables

|  | **ICD-9** | **ICD-10** | **Birth certificate indication** |
| --- | --- | --- | --- |
| Infant codes |  |  |  |
| Bronchopulmonary dysplasia | 770.7 | P27.1 |  |
| Necrotizing enterocolitis | 777.5 | P77 |  |
| Intraventricular hemorrhage > grade II | 772.13, 772.14 | P52.2 |  |
| Periventricular leukomalacia | 779.7 | P91.2 |  |
| Retinopathy of prematurity > stage 2 | 362.25-7 or procedure codes 14.2, 14.5, 14.7 14.9 | H35.14-6 any procedure code for surgery on retina or choroid plexus |  |
| Maternal codes |  |  |  |
| Preeclampsia | 642.4, 642.5, 642.6, 642.7 | O11, O14.0, O14.1, O14.2, O14.9, O15, O16 | X |
| Hypertension | 642.0, 642.1, 642.2, 642.3, 642.9  Infant code 760.0 | O10, O13  Infant code: P00.0 | X |
| Diabetes | 648.0, 648.8, 249, 250 | E10, E11, E12, E13, E14, O24.0, O24.1, O24.2, O24.3, O24.4, O24.9  Infant code: P70.0, P70.1 | X |
| Drug abuse | 304, 305.2, 305.3, 305.4, 305.5, 305.6, 305.7, 305.8, 305.9, 648.3 | F11, F12, F13, F14, F15, F16, F18, F19  Infant code: P04.4 | . |
| Infection during pregnancy | 646.5, 646.6, 647  Infant code: 760.1, 760.2 | O23.0, O23.1, O23.2, O23.3, O23.4, O98  Infant code P00.1, P00.2 | . |
| Placental abruption | 641.2  Infant codes: 762.1 | O45  Infant codes: P02.1 |  |
| Uterine rupture | 665.0, 665.1 | O71.0, O71.1 | . |
| Preterm premature rupture of the membranes | Gestation < 37 weeks and 658.1  Infant code: 761.1 | Gestation < 37 weeks and O42  Infant code: P01.1 | X |
| Cesarean delivery | 669.7 or procedure 74  Infant code: 763.4 | O82 or procedure 10D00Z0, 10D00Z1, 10D00Z2  Infant code: P03.4 | X |
| Oligohydramnios | 658.0  Infant code: 761.2 | O41.0  Infant code: P01.2 |  |
| Polyhydramnios | 657  Infant code: 761.3 | O40  Infant code: P01.3 |  |
| Premature labor | 644 | O60 | X |
| Smoking | 305.1, 649.0 | F17.2, Z72.0  Infant code P04.2 | X |
