# Supplementary material for: Developing a resiliency model for survival without major morbidity in preterm infants
Source: J Perinatol. 2022 Oct 11;43(4):452–7. doi: 10.1038/s41372-022-01521-3 (PMC10079534; doi:10.1038/s41372-022-01521-3)
Supplement: Supplementary file 3 — supplemental Table 3 [file 41372_2022_1521_MOESM3_ESM.docx]

Supplemental Table 3: National Birth Defects Registry Definition of Major Birth Defects and chromosomal anomalies used in Iowa.

| **Birth Defect** | **ICD-9-CM Codes** | **ICD-10-CM Codes** |
| --- | --- | --- |
| **CNS** |  |  |
| **Anencephalus** | 740.0x — 740.1x | Q00.0x — Q00.1x |
| **Spina bifida without**  **anencephalus** | 741.0x, 741.9x w/o 740.0x, 740.10x | Q05.x, Q07.01, Q07.03 |
| **Encephalocele** | 742.0x | Q01.x |
| **Holoprosencephaly** | 742.2x | Q04.2 |
| **Eye** |  |  |
| **Anophthalmia/microphthalmia** | 743.0x, 743.1x | Q11.0x — Q11.2x |
| **Congenital cataract** | 743.30x — 743.34x | Q12.0x |
| **Ear** |  |  |
| **Anotia/microtia** | 744.01x, 744.23x | Q16.0x, Q17.2x |
| **Cardiovascular** |  |  |
| **Aortic valve stenosis** | 746.3x | Q23.0x |
| **Atrioventricular septal defect (endocardial cushion defect)** | 745.60x, 745.61x, 745.69x | Q21.2x |
| **Coarctation of the aorta** | 747.10x | Q25.1x |
| **Common truncus (truncus arteriosus or TA)** | 745.0x | Q20.0x |
| **Double outlet right ventricle (DORV)** | 745.11x | Q20.1x |
| **Ebstein anomaly** | 746.2x | Q22.5x |
| **Hypoplastic left heart syndrome** | 746.7x | Q23.4x |
| **Interrupted aortic arch (IAA)** | 747.11x | Q25.2x, Q25.4x |
| **Pulmonary valve atresia and stenosis** | 746.01x (atresia),  746.02x (stenosis) | Q22.0x, Q22.1x |
| **Single Ventricle** | 745.3x | Q20.4x |
| **Tetralogy of Fallot (TOF)** | 745.2x | Q21.3x |
| **Total anomalous pulmonary venous connection (TAPVC)** | 747.41x | Q26.2x |
| **Transposition of the great arteries (TGA)** | 745.10x, 745.12x, 745.19x | Q20.3x, Q20.5x |
| **Tricuspid value atresia and stenosis** | 746.1x | Q22.4x |
| **Orofacial** |  |  |
| **Choanal atresia** | 748.0x | Q30.0x |
| **Cleft lip with cleft palate** | 749.2x | Q37.x |
| **Cleft lip alone (without cleft palate)** | 749.1x | Q36. x |
| **Cleft palate alone (without cleft lip)** | 749.0x | Q35.x |
| **Gastrointestinal** |  |  |
| **Biliary atresia** | 751.61x | Q44.2x — Q44.3x |
| **Esophageal atresia/tracheoesophageal fistula** | 750.3x | Q39.0x — Q39.4x |
| **Rectal and large intestinal atresia/stenosis** | 751.2x | Q42.x |
| **Small intestinal atresia/stenosis** | 751.1x | Q41.x |
| **Genitourinary** |  |  |
| **Bladder exstrophy** | 753.5x | Q64.10x, Q64.19x |
| **Cloacal exstrophy** | 751.5x | Q64.12x |
| **Congenital Posterior Urethral Valves** | 753.6x | Q64.2x |
| **Hypospadias** | 752.61x | Q54.0x—Q54.9x  Excluding Q54.4x |
| **Renal agenesis/hypoplasia** | 753.0x | Q60.0x — Q60.6x |
| **Musculoskeletal** |  |  |
| **Clubfoot** | 754.51x, 754.70x | Q66.0x, Q66.89x |
| **Craniosynostosis** | No specified code | Q75.0x |
| **Diaphragmatic hernia** | 756.6x | Q79.0x, Q79.1x |
| **Gastroschisis** | 756.73x, 756.79x | Q79.3x |
| **Limb deficiencies (reduction defects)** | 755.2x — 755.4x | Q71.x, Q72.x, Q73.x |
| **Omphalocele** | 756.72, 756.79x | Q79.2x |
| **Chromosomal** |  |  |
| **Deletion 22 q11** | 758.32x | Q93.81x |
| **Trisomy 13** | 758.1x | Q91.4x — Q91.7x |
| **Trisomy 18** | 758.2x | Q91.0x — Q91.3x |
| **Trisomy 21 (Down syndrome)** | 758.0x | Q90.x |
| **Turner Syndrome** | 758.6x | Q96.x |

NOTE: x means included all decimal places after this (e.g. for Encephalocele: code is 742.0x, so individuals with 742.0, 742.01, 742.02, 742.03, 742.04, etc. would be counted as having this major congenital anomaly)
