# Supplementary material for: Developing a resiliency model for survival without major morbidity in preterm infants
Source: J Perinatol. 2022 Oct 11;43(4):452–7. doi: 10.1038/s41372-022-01521-3 (PMC10079534; doi:10.1038/s41372-022-01521-3)
Supplement: Supplementary file 4 — supplemental Table 4 [file 41372_2022_1521_MOESM4_ESM.docx]

**Supplemental Table 4:** Calibration for validation sample for survival (in deciles)

|  | **Survival** | | **Survival without major morbidity** | |
| --- | --- | --- | --- | --- |
| **n** | **Observed**  **(%)** | **Predicted**  **(%)** | **Observed**  **(%)** | **Predicted**  **(%)** |
| 669 | 28.0 | 28.3 | 6.6 | 7.8 |
| 668 | 72.2 | 73.8 | 27.4 | 28.6 |
| 668 | 87.7 | 87.7 | 48.4 | 47.6 |
| 668 | 93.8 | 93.3 | 65.8 | 65.2 |
| 668 | 95.5 | 95.9 | 78.1 | 78.6 |
| 669 | 96.9 | 97.8 | 85.5 | 85.7 |
| 671 | 98.4 | 98.3 | 89.7 | 90.0 |
| 665 | 98.5 | 98.7 | 92.8 | 92.9 |
| 669 | 98.2 | 98.7 | 94.3 | 94.9 |
| 667 | 98.5 | 99.1 | 95.7 | 96.5 |
