# Supplementary material for: Developing a resiliency model for survival without major morbidity in preterm infants
Source: J Perinatol. 2022 Oct 11;43(4):452–7. doi: 10.1038/s41372-022-01521-3 (PMC10079534; doi:10.1038/s41372-022-01521-3)
Supplement: Supplementary file 5 — supplemental Table 5 [file 41372_2022_1521_MOESM5_ESM.docx]

**Supplemental Table 5:** In-sample predicted probability for outcomes based on all covariates by gestational age

|  | **Observed predicted survival range** | | **Observed predicted range of survival without major morbidity** | |
| --- | --- | --- | --- | --- |
| **GA (weeks)** | **Smallest** | **Largest** | **Smallest** | **Largest** |
| 22 | 0.07 | 23.1 | 0.1 | 6.3 |
| 23 | 0.5 | 66.7 | 0.4 | 20.0 |
| 24 | 1.7 | 87.4 | 1.2 | 42.8 |
| 25 | 4.0 | 94.4 | 2.5 | 60.1 |
| 26 | 6.5 | 96.5 | 4.1 | 71.6 |
| 27 | 13.4 | 98.4 | 7.6 | 84.0 |
| 28 | 17.6 | 98.9 | 13.6 | 89.8 |
| 29 | 23.3 | 99.2 | 22.7 | 94.7 |
| 30 | 31.6 | 99.5 | 34.2 | 97.0 |
| 31 | 39.9 | 99.6 | 46.8 | 98.3 |
