# Supplementary material for: Developing a resiliency model for survival without major morbidity in preterm infants
Source: J Perinatol. 2022 Oct 11;43(4):452–7. doi: 10.1038/s41372-022-01521-3 (PMC10079534; doi:10.1038/s41372-022-01521-3)
Supplement: Supplementary file 6 — supplemental Table 6 [file 41372_2022_1521_MOESM6_ESM.docx]

**Supplemental Table 6:** Observed to expected outcome probabilities for sociodemographic groups

|  | Observed rate  (%) | Predicted rate based on score*  (%) |
| --- | --- | --- |
| **A) Survival** | | |
| Race/ethnicity |  |  |
| White | 87.0 | 88.2 |
| Black | 85.3 | 84.7 |
| Asian | 88.2 | 88.8 |
| Hispanic | 86.9 | 87.1 |
| Other | 81.0 | 83.9 |
| Insurance status |  |  |
| Public | 85.8 | 86.6 |
| Private | 87.5 | 87.6 |
| Self-pay | 83.4 | 84.5 |
| Other | 87.9 | 87.7 |
| Maternal Education |  |  |
| < 12 years | 86.9 | 87.1 |
| = 12 years | 84.5 | 86.0 |
| > 12 years | 87.7 | 87.7 |
| **B) Survival without morbidity** | | |
| Race/ethnicity |  |  |
| White | 68.7 | 70.7 |
| Black | 65.1 | 64.5 |
| Asian | 70.2 | 70.8 |
| Hispanic | 69.4 | 69.2 |
| Other | 62.4 | 65.2 |
| Insurance status |  |  |
| Public | 67.8 | 68.4 |
| Private | 68.8 | 69.5 |
| Self-pay | 69.7 | 67.5 |
| Other | 72.8 | 71.0 |
| Maternal Education |  |  |
| < 12 years | 69.5 | 69.3 |
| = 12 years | 67.1 | 67.7 |
| > 12 years | 68.9 | 69.6 |

*mean value of predicted outcome by group
