# Supplementary material for: Developing a resiliency model for survival without major morbidity in preterm infants
Source: J Perinatol. 2022 Oct 11;43(4):452–7. doi: 10.1038/s41372-022-01521-3 (PMC10079534; doi:10.1038/s41372-022-01521-3)
Supplement: Supplementary file 7 — supplemental Figure 1 [file 41372_2022_1521_MOESM7_ESM.docx]

**Supplemental Figure 1:** CV plots for training sample
