# Supplementary material for: Developing a resiliency model for survival without major morbidity in preterm infants
Source: J Perinatol. 2022 Oct 11;43(4):452–7. doi: 10.1038/s41372-022-01521-3 (PMC10079534; doi:10.1038/s41372-022-01521-3)
Supplement: Supplementary file 8 — supplemental Figure 2 [file 41372_2022_1521_MOESM8_ESM.docx]

**Supplemental Figure 2:** ROC for internal validation sample

ROC for survival 0.895 (95% CI 0.882-0.908)

ROC for survival without major morbidity 0.867 (95% CI 0.857-0.877)
